# Mechanical ventilation causes diaphragm dysfunction in newborn lambs

**DOI:** 10.1186/s13054-019-2409-6

**Published:** 2019-04-16

**Authors:** Feng Liang, Guillaume Emeriaud, Dilson E. Rassier, Dong Shang, Ekaterina Gusev, Sabah N. A. Hussain, Michael Sage, Benjamin Crulli, Etienne Fortin-Pellerin, Jean-Paul Praud, Basil J. Petrof

**Affiliations:** 1 Meakins-Christie Laboratories and Translational Research in Respiratory Diseases Program, McGill University Health Centre and Research Institute, 1001 Decarie Boulevard, Montreal, QC H4A 3J1 Canada; 2 Pediatric Intensive Care Unit, Department of Pediatrics, Sainte-Justine Hospital, University of Montreal, Montreal, QC Canada; 3 Department of Kinesiology, McGill University, Montreal, QC Canada; 4 Neonatal Respiratory Research Unit, Department of Pediatrics, University of Sherbrooke, Sherbrooke, QC Canada

**Keywords:** Mechanical ventilation, Ventilator-induced diaphragmatic dysfunction (VIDD), Neonatal, Surfactant deficiency, Lung injury

## Abstract

**Background:**

Diaphragm weakness occurs rapidly in adult animals treated with mechanical ventilation (MV), but the effects of MV on the neonatal diaphragm have not been determined. Furthermore, it is unknown whether co-existent lung disease exacerbates ventilator-induced diaphragmatic dysfunction (VIDD). We investigated the impact of MV (mean duration = 7.65 h), either with or without co-existent respiratory failure caused by surfactant deficiency, on the development of VIDD in newborn lambs.

**Methods:**

Newborn lambs (1–4 days) were assigned to control (CTL, non-ventilated), mechanically ventilated (MV), and MV + experimentally induced surfactant deficiency (MV+SD) groups. Immunoblotting and quantitative PCR assessed inflammatory signaling, the ubiquitin-proteasome system, autophagy, and oxidative stress. Immunostaining for myosin heavy chain (MyHC) isoforms and quantitative morphometry evaluated diaphragm atrophy. Contractile function of the diaphragm was determined in isolated myofibrils ex vivo.

**Results:**

Equal decreases (25–30%) in myofibrillar force generation were found in MV and MV+SD diaphragms compared to CTL. In comparison to CTL, both MV and MV+SD diaphragms also demonstrated increased STAT3 transcription factor phosphorylation. Ubiquitin-proteasome system (Atrogin1 and MuRF1) transcripts and autophagy indices (Gabarapl1 transcripts and the ratio of LC3B-II/LC3B-I protein) were greater in MV+SD relative to MV alone, but fiber type atrophy was not observed in any group. Protein carbonylation and 4-hydroxynonenal levels (indices of oxidative stress) also did not differ among groups.

**Conclusions:**

In newborn lambs undergoing controlled MV, there is a rapid onset of diaphragm dysfunction consistent with VIDD. Superimposed lung injury caused by surfactant deficiency did not influence the severity of early diaphragm weakness.

**Electronic supplementary material:**

The online version of this article (10.1186/s13054-019-2409-6) contains supplementary material, which is available to authorized users.

## Background

Pulmonary diseases are among the most important causes of pediatric morbidity and mortality during the neonatal period. Despite tremendous advances in perinatal care, surfactant deficiency remains a major cause of respiratory compromise at birth, often requiring the use of mechanical ventilation (MV). MV is life-saving in this context, but also has the potential to exacerbate acute or long-term lung injury [1, 2]. In addition, there is increased recognition of the adverse effects of MV not only on the lungs, but also upon the diaphragm, a condition referred to as ventilator-induced diaphragmatic dysfunction (VIDD) [3]. Multiple studies in adult animals have shown that controlled MV (i.e., without significant spontaneous respiratory efforts) leads to an early decrease in the force-generating capacity of diaphragm muscle fibers, which is then followed by the development of diaphragm fiber atrophy [3, 4]. The evidence for VIDD in humans, derived primarily from adult patients with acute respiratory failure [5–8], is consistent with these animal model data [9].

Few studies have analyzed the impact of MV on the diaphragm during the first few days of post-natal life. Like the lungs, the diaphragm undergoes major changes at birth, particularly with respect to its contractile protein composition and metabolic profile [10]. In neonates, the post-inspiratory activity of the diaphragm also helps to maintain end-expiratory lung volume [11]. However, in critically ill children, a complete cessation of diaphragm electromyographic activity is frequently observed during MV [12]. Taken together, these observations suggest that neonates could be at especially high risk for the development of VIDD. In addition, a frequent indication for MV in neonatal intensive care is the presence of lung injury secondary to surfactant deficiency, which could in theory further exacerbate diaphragm weakness via increased local or systemic inflammation [13–15].

In the present study, we employed a well-established newborn lamb model [16] to elucidate the effects of MV on the neonatal diaphragm. Our primary objective was to establish whether MV during the early post-natal period leads to a loss of diaphragmatic force-generating capacity. We also examined inflammatory signaling (e.g., STAT3 phosphorylation), induction of muscle proteolysis (e.g., via the ubiquitin-proteasome and autophagy pathways), and oxidative stress, which have all been reported to play a role in VIDD pathogenesis [3, 4]. Our second major objective was to ascertain whether lung injury caused by pulmonary surfactant deficiency, which occurs in the setting of prematurity, can increase the severity of VIDD in newborn lambs.

## Methods

### Animals and mechanical ventilation

Newborn male lambs (1–4 days old) delivered at term were randomly assigned to one of three groups: (1) control animals (CTL, *n* = 5), which were immediately euthanized by an IV injection of pentobarbital (90 mg/kg) without being subjected to MV; (2) mechanically ventilated animals (MV, *n* = 6); and (3) mechanically ventilated animals with surfactant depletion (MV+SD, *n* = 6). Surfactant depletion was induced by serial intrapulmonary isotonic saline lavage (median of seven aliquots of 10 ml/kg saline) until a target reduction of the PaO2/FiO2 ratio (< 100) was achieved [17]. Mechanically ventilated lambs were anesthetized (ketamine 1 mg/kg/h, propofol 6–10 mg/kg/h IV), and paralyzed (rocuronium 0.6 mg/kg IV) to achieve appropriate sedation and prevent spontaneous breathing. A lung-protective protocol (target tidal volume = 6–8 ml/kg, PaCO2 = 45–55 mmHg, SaO2 = 90–100%) was employed for a mean duration of 7.65 + 0.37 h (SE) in the dorsal position. All mechanically ventilated animals survived the protocol. After euthanasia, the diaphragm was immediately exposed and full thickness biopsies were obtained from the lateral costal portion of the muscle midway between the ribcage and the central tendon. The study was approved by the animal research ethics board of the University of Sherbrooke (protocol # 423-17B) and performed in accordance with the Canadian Council on Animal Care.

### Immunoblotting

Protein was extracted from frozen diaphragm samples, and antibody dilutions were selected according to the manufacturers’ instructions. The following proteins were analyzed as previously described: (1) total and phosphorylated (Tyr705) forms of STAT3 (clones 124H6 and D3A7, Cell Signaling, USA); (2) carbonylated proteins (Oxyblot Protein Oxidation Detection Kit, Millipore, Germany) and 4-hydroxynonenal (4-HNE; R&D Systems, USA) as indices of oxidative stress; and (3) LC3B-I and LC3B-II levels (clone D11, Cell Signaling, USA) as indices of autophagy [8, 18, 19]. Immunoreactive bands were visualized using enhanced chemiluminescence and Ponceau red for protein loading. Band intensities were quantified using the Odyssey Infrared Imaging System (LI-COR Biosciences, USA), and all values are expressed as *n*-fold relative to the mean CTL group value.

### Quantitative PCR

Relative mRNA transcript levels were determined with normalization to a combination of three housekeeping genes (glyceraldehyde-3-phosphate dehydrogenase (GAPDH), ribosomal protein L19 (RPL19), and RNA polymerase polypeptide A (POLR2A)). Primer sequences for genes involved in STAT3 activation (IL-6), the ubiquitin-proteasome pathway of proteolysis (Atrogin1, MuRF1), metabolism (SIRT1), autophagy (LC3B, Gabarapl1), and myosin heavy chain (MyHC) isoforms (1, 2a, 2x, embryonic, neonatal) are shown in Additional file 1: Table S1. The data are expressed as *n*-fold change relative to the average CTL group value according to the standard ΔΔ^CT^ method [8, 18, 19].

### Diaphragm morphometry

Cryostat sections (8 μm thick) were stained with antibodies (all from Developmental Hybridoma Bank, USA) against slow-twitch type 1 (BA-D5, 1:25), fast-twitch type 2a (SC-71, 1:300) and fast-twitch type 2x (6H1, 1:25) MyHC isoforms as we have previously described [20]. Images were acquired with an AxioCam MRm camera and stitched using Zen Blue software. The quantitative analysis of MyHC fiber type proportions, as well as the size of individual fibers using Feret’s minimal diameter (to avoid inaccuracies related to oblique tissue sectioning), was determined on at least 200 fibers per sample using ImageJ.

### Diaphragm myofibrillar contractility

The following measurements were made on diaphragm biopsies using methods we have described in detail [21]: (1) maximal isometric force generation, (2) rate of force development (Kact), (3) rate of force redevelopment after acute shortening (Ktr), and (4) rate of relaxation (Krel). Briefly, diaphragm muscle bundles were dissected, chemically permeabilized, and homogenized to isolate myofibrils. Myofibrils were attached at one end to an atomic force microscope cantilever (model ATEC-CONTPT, Nanosensors, USA) and at the other end to a glass microneedle connected to a piezoelectric motor. When myofibrils contract, deflection of the cantilever is detected by a laser beam and the force generated is then determined based on its displacement. A multichannel perfusion system was used to manipulate calcium concentrations, thereby inducing muscle contraction and relaxation.

### Statistics

Data are expressed as mean values ± SE and experimental groups were compared by one-way ANOVA after log transformation to normalize the data distribution [22]. The Tukey test was used to adjust for multiple comparisons (GraphPad Prism, La Jolla, CA, USA) unless stated otherwise. The study was powered (1 − *β* = 0.80) to detect a 30% decrease in the primary outcome (diaphragm force) following MV, based on our previous data using the same method in humans [21]. Statistical significance was set at *p* < 0.05 for all tests.

## Results

The mean age of the animals was 2.6 + 0.3 days and did not differ among the three groups. The total duration of ventilation was longer in the surfactant-depleted lambs after taking into account the additional time required for intrapulmonary lavage (MV = 6.80 + 0.16 h, MV+SD = 8.55 + 0.57 h; *p* < 0.05). The MV settings and respiratory parameters in the MV and MV+SD lambs are shown in Table 1. As shown, the MV+SD group had significantly reduced dynamic compliance as well as higher oxygen requirements and a lower PaO2/FiO2 ratio, consistent with pulmonary dysfunction induced by surfactant depletion [17].

**Table 1:** Ventilator settings and gas exchange parameters in the two groups of newborn lambs receiving mechanical ventilation

Respiratory parameters	MV (*N* = 6)	MV+SD (*N* = 6)
Respiratory rate (bpm)	60 (59–60)	53 (47–56)^a^
Tidal volume (ml/kg)	7.8 (7.7–7.9)	5.9 (5.7–6.6)^a^
FiO_2_ (%)	25 (22–25)	71 (60–95)^a^
PEEP (cmH_2_O)	6.0 (5.3–6.0)	6.0 (5.3–6.0)
Peak inspiratory airway pressure (cmH_2_O)	16.0 (15.0–16.0)	17.0 (14.8–18.5)
Arterial pH	7.33 (7.31–7.37)	7.26 (7.16–7.28)^b^
PaO_2_ (mmHg)	63 (52–67)	97 (92–110)^a^
PaCO_2_ (mmHg)	46 (45–50)	48 (39–49)
PaO_2_/FiO_2_ ratio	271 (232–283)	142 (105–161)^a^
Dynamic compliance (ml/kg/cmH_2_O)	0.77 (0.68–0.79)	0.58 (0.48–0.69)^a^

Dynamic compliance = tidal volume/peak inspiratory airway pressure minus PEEP. Data are reported as median (interquartiles) after 5 h of mechanical ventilation

*PEEP* positive end-expiratory pressure, *FiO2* fraction of inspired oxygen

^a^*p* < 0.01 or ^b^*p* < 0.05 (rank test)

### Inflammatory signaling

**Fig. 1:**
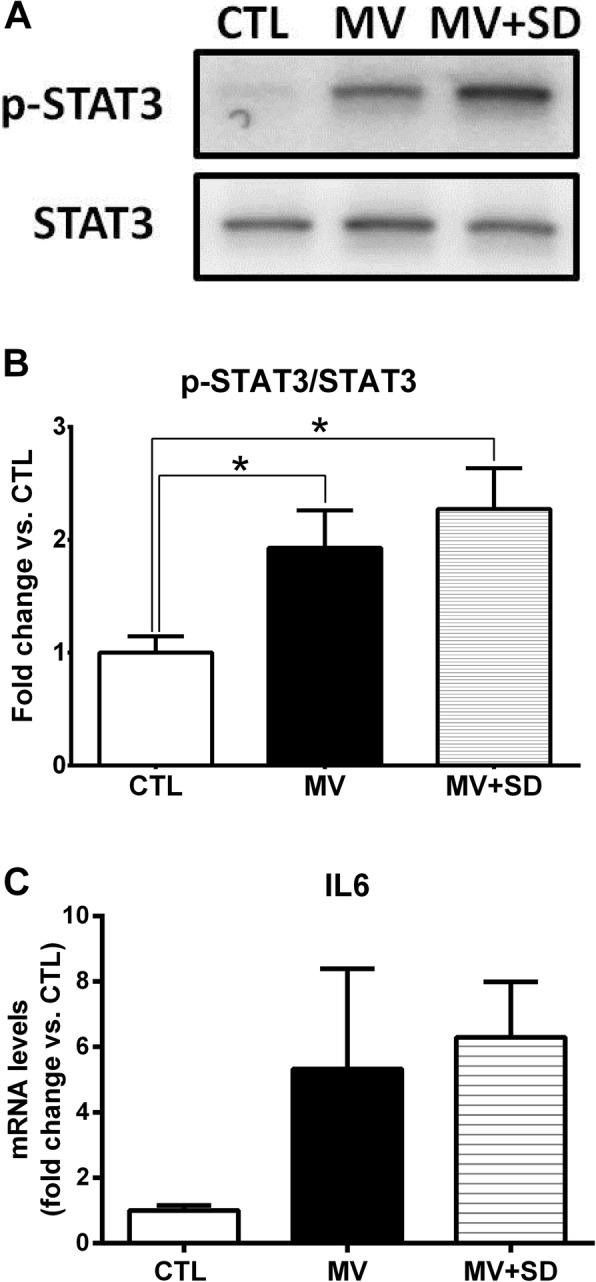
Inflammatory signaling in mechanically ventilated newborn diaphragms. **a** Representative immunoblot images of phosphorylated and total STAT3 protein in the diaphragm. **b** Group mean ratio of phosphorylated to total STAT3 protein in the diaphragm, expressed as fold change relative to CTL. **c** Group mean transcript levels of IL-6, depicted as fold change relative to CTL group values. *p < 0.05 for CTL (*n* = 5) versus MV (*n* = 6) or MV+SD (*n* = 6)

MV and MV+SD diaphragms exhibited significantly increased STAT3 phosphorylation relative to the CTL group (Fig. 1a, b) but did not differ from one another in this respect. Mean transcript levels of IL-6, a prototypical upstream inducer of STAT3 activation, were also elevated in the two mechanically ventilated groups although these changes did not achieve statistical significance versus CTL (Fig. 1c).

### Proteolysis pathways

**Fig. 2:**
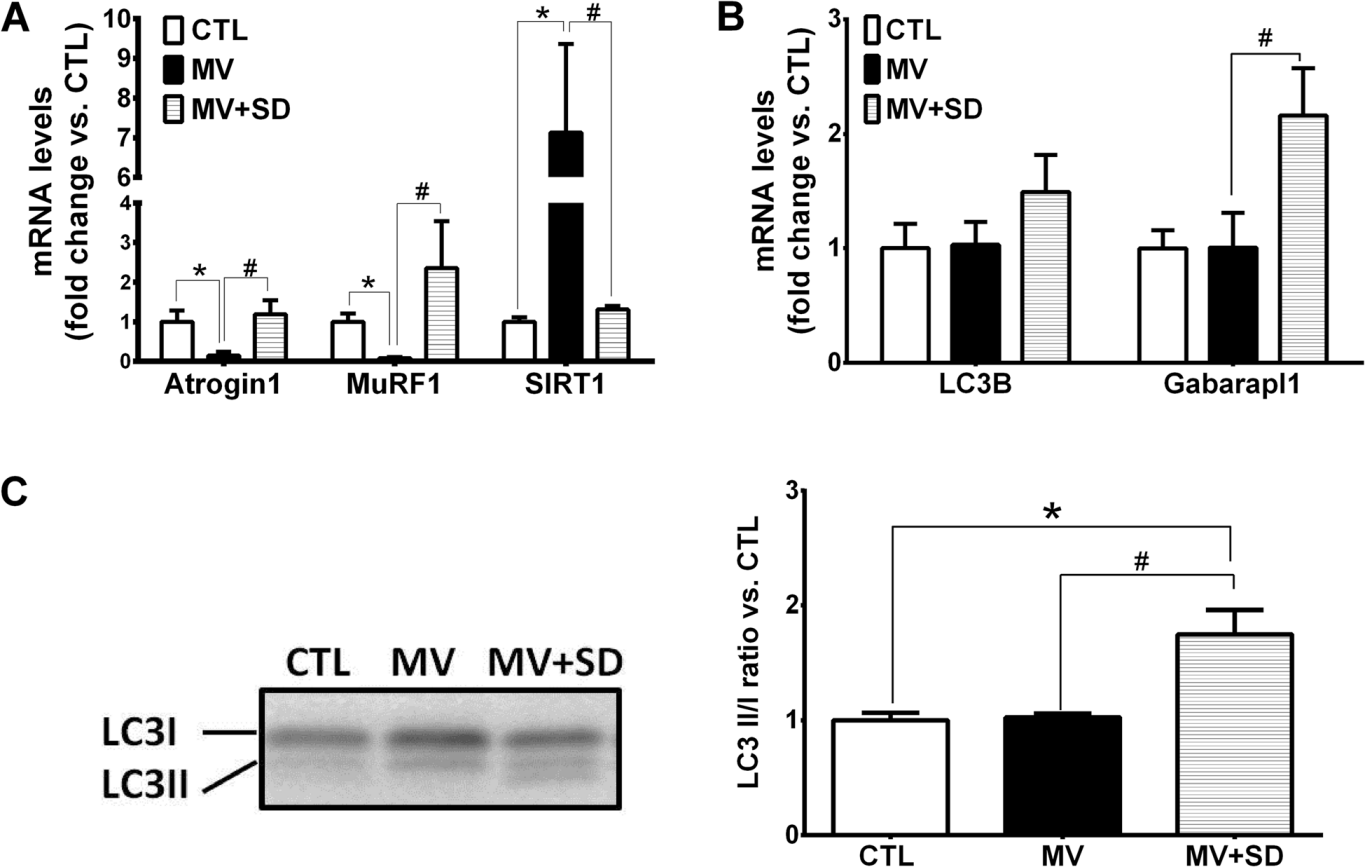
Proteolysis pathways in mechanically ventilated newborn diaphragms. **a** Transcript levels of muscle-specific E3 ubiquitin ligases (Atrogin1, MuRF1) and SIRT1 in the diaphragm. **b** Transcript levels of autophagy genes LC3B and Gabarapl1, expressed as fold change relative to CTL values. **c** Representative immunoblot and group mean values for LC3B-I and LC3B-II protein in the diaphragm. *p < 0.05 versus CTL; ^#^*p* < 0.05 for MV versus MV+SD

To assess proteolysis through the ubiquitin-proteasome system (UPS), transcript levels of the two muscle-specific E3 ubiquitin ligases, Atrogin1 and MuRF1, were determined. Atrogin1 and MuRF1 mRNA levels were significantly decreased in the MV group in comparison to CTL diaphragms (Fig. 2a). This was not observed in the MV+SD diaphragms, which did not differ from the CTL group. Conversely, transcript levels of Sirtuin (SIRT) 1, a histone deacetylase previously implicated in the regulation of UPS-mediated proteolysis, were markedly increased in the MV group in comparison to both CTL and MV+SD diaphragms.

To evaluate autophagy activation in the diaphragm, transcript levels of prototypical autophagy pathway genes (LC3B, Gabarapl1) were first determined. At the mRNA level, Gabarapl1 expression was significantly increased in the MV+SD diaphragms compared to the other groups and there was a similar trend toward greater LC3B expression (Fig. 2b). Furthermore, protein levels of the non-lipidated (LC3B-I) and lipidated (LC3B-II) forms of LC3B revealed that the LC3B-II/LC3B-I ratio, considered an index of autophagy activation, was increased in the MV+SD group (Fig. 2c). Taken together, these results suggest that MV+SD may have led to a higher level of autophagy pathway induction compared to the other groups.

### Oxidative stress

**Fig. 3:**
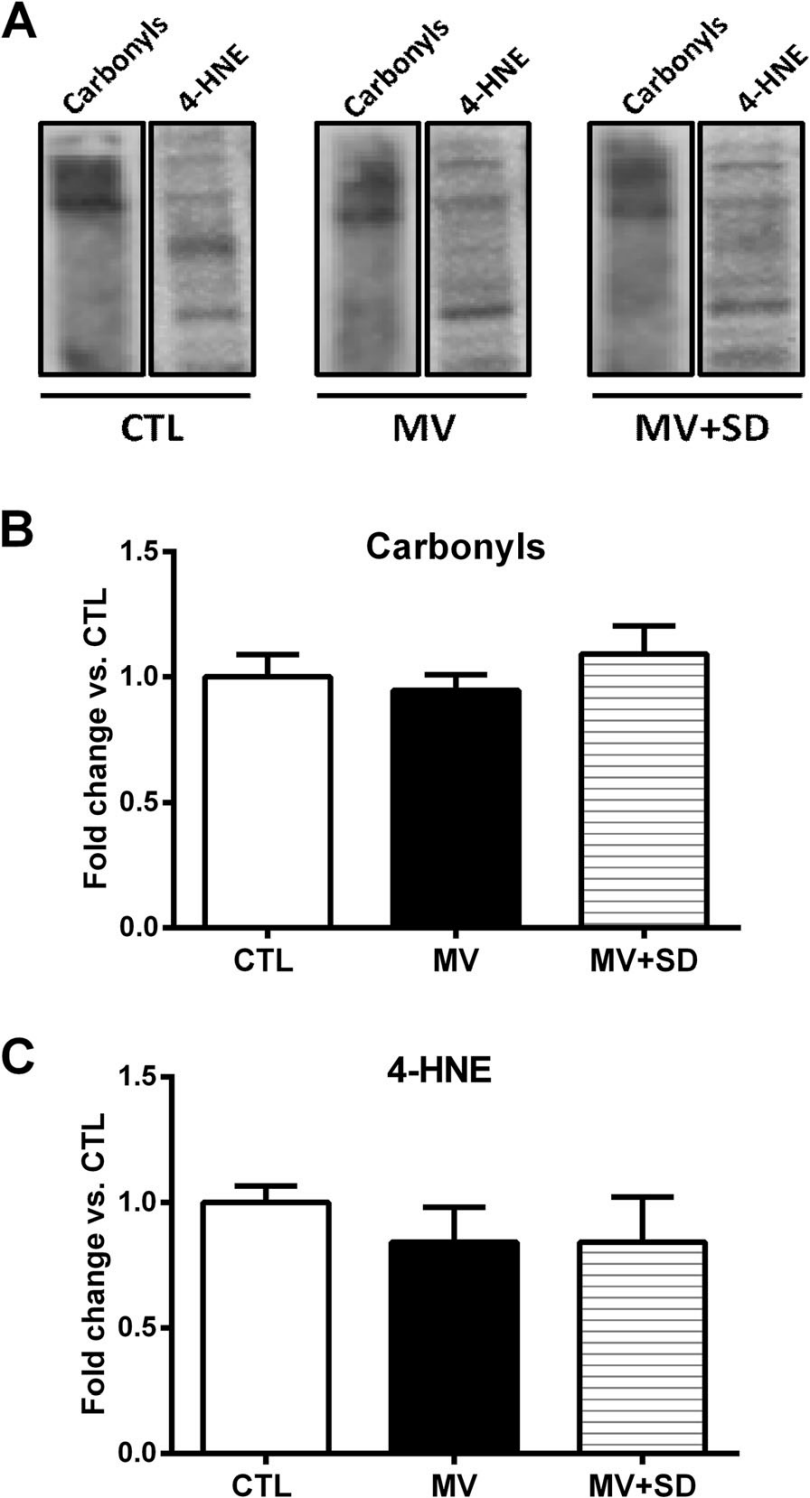
Indices of oxidative stress in mechanically ventilated newborn diaphragms. **a** Representative immunoblot images of carbonyl groups and 4-hydroxynonenal (4-HNE) in diaphragm proteins extracted from CTL, MV, and MV+SD groups. Group mean quantification of **b** carbonyls and **c** 4-HNE in the diaphragm under the above experimental conditions

**Fig. 4:**
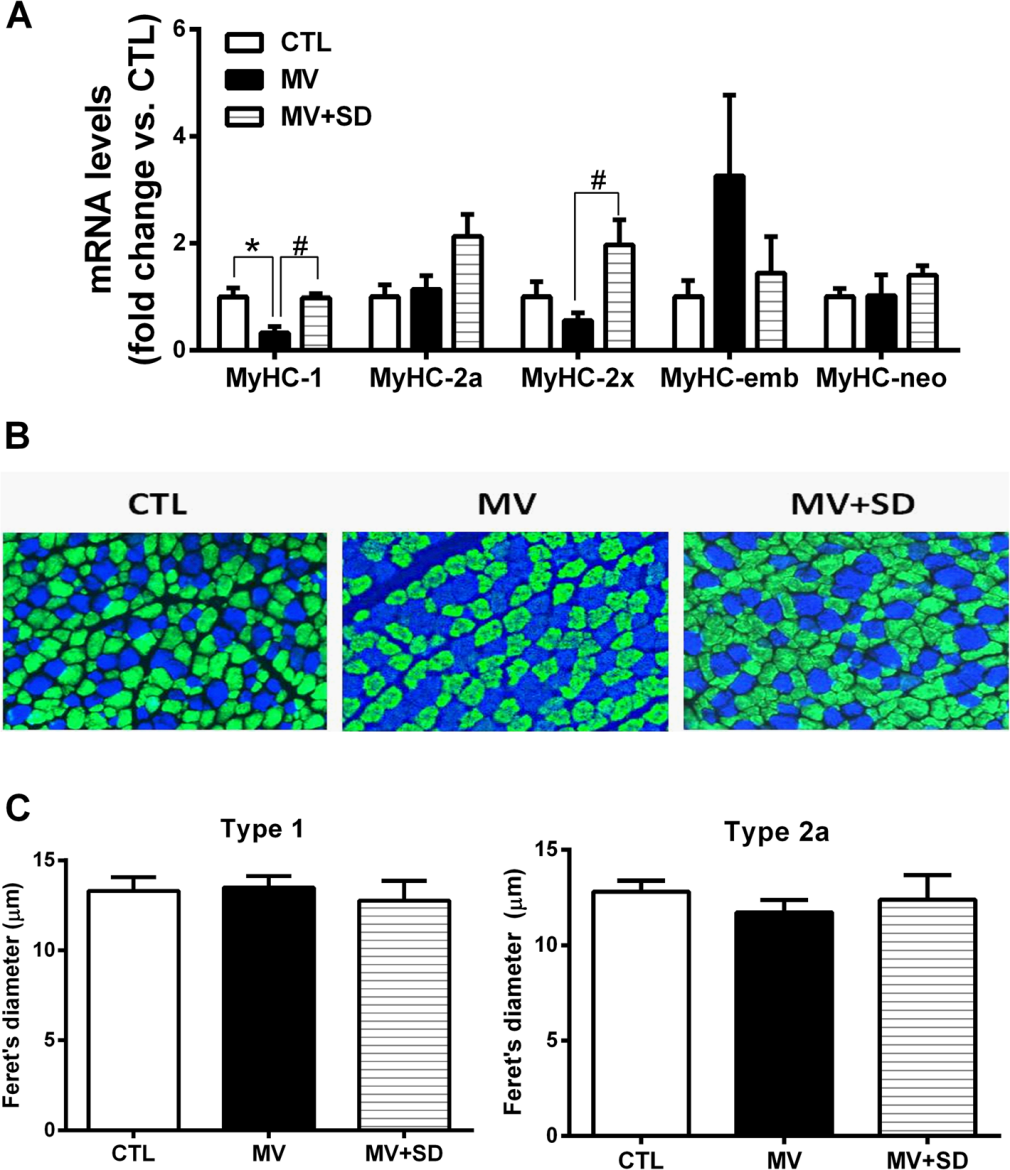
Diaphragm muscle fiber phenotype in mechanically ventilated newborn lambs. **a** Transcript levels of MyHC isoforms in the diaphragm. **b** Representative immunohistochemical staining for slow type 1 (blue) and fast type 2a (green) MyHC isoforms. **c** Group mean diaphragm muscle fiber size (Feret’s minimal diameter) for type 1 and type 2a fibers in the three experimental groups. *p < 0.05 versus CTL; ^#^*p* < 0.05 for MV versus MV+SD

The effects of MV and MV+SD on oxidative stress in the diaphragm were determined by quantifying protein carbonylation (Fig. 3a) and 4-HNE adducts (an index of lipid peroxidation) in the muscle (Fig. 3b). Neither of these two parameters showed any significant differences among the three experimental groups.

### Myosin heavy chain (MyHC) gene expression and muscle fiber phenotype

Expression levels of the adult (types 1, 2a, 2x) and developmental (embryonic, neonatal) isoforms of MyHC were quantified at the transcript level in whole diaphragm tissue. Type 1 MyHC expression was decreased in the MV diaphragms, whereas type 2x MyHC was mildly upregulated in the MV+SD group (Fig. 4a). There were no other significant effects of either MV or MV+SD on MyHC isoform gene expression.

Immunohistochemistry determined the MyHC phenotype at the level of individual fibers, classified according to the predominant MyHC isoform expressed. Type 1 and type 2a fibers essentially constituted the entirety of the diaphragm myofiber population (Fig. 4b). Despite the presence of type 2x mRNA, the corresponding protein could not be detected. The relative proportions of type 1 and 2a fibers were unaltered in the MV (type 1 = 32.2 ± 1.4%; type 2a = 67.8 ± 1.3%) and MV+SD (type 1 = 30.8 ± 0.9%; type 2a = 69.2 ± 0.9%) diaphragms in comparison to the CTL group (type 1 = 32.9 ± 0.9%; type 2a = 67.1 ± 0.8%). In addition, quantification of fiber size in type 1 and type 2a fiber populations did not reveal significant differences between the groups (Fig. 4c). Standard hematoxylin and eosin staining also did not show signs of muscle damage or impaired regeneration, such as necrosis or centrally nucleated fibers.

### Diaphragm myofibril contractile performance

**Fig. 5:**
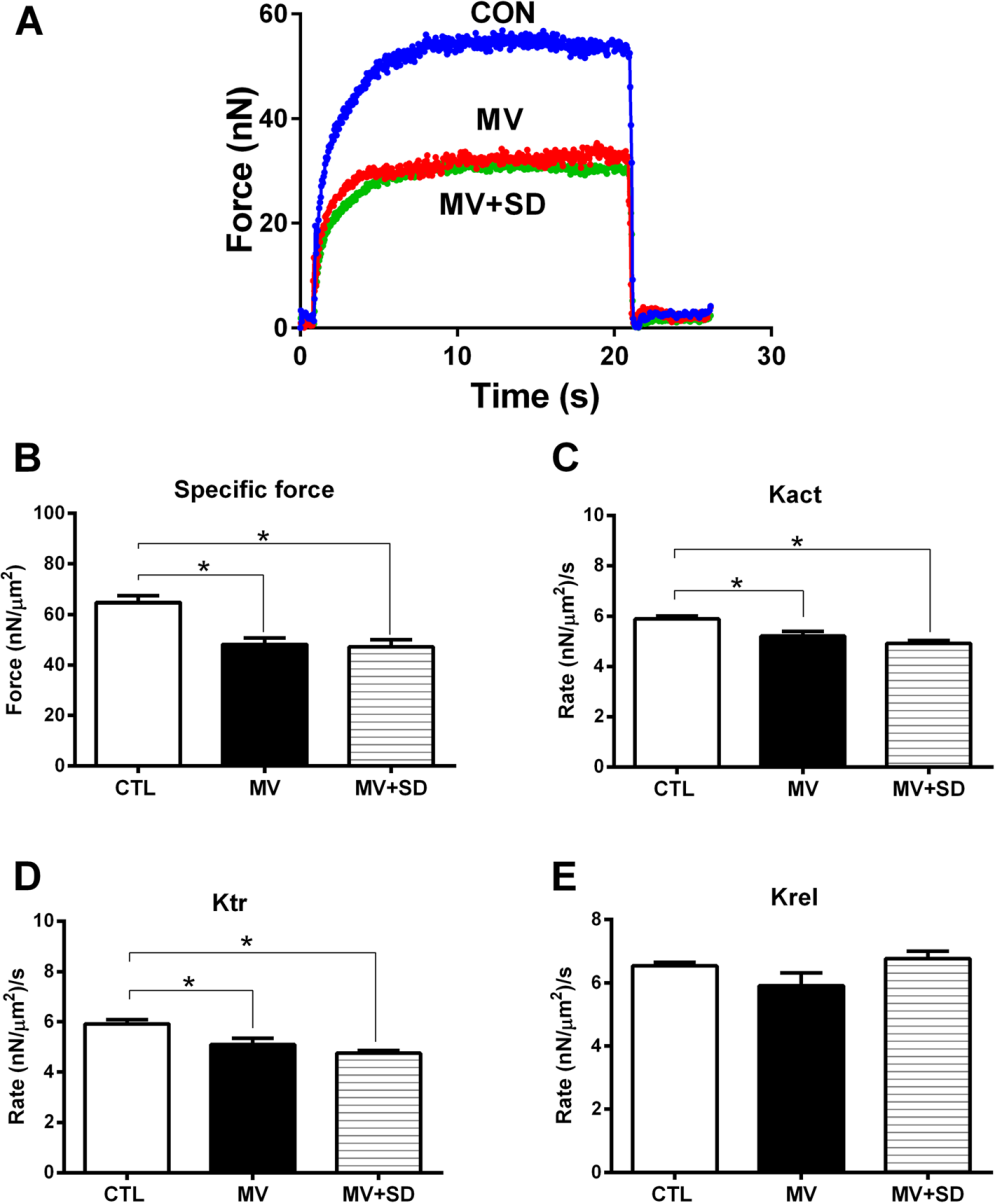
Diaphragm muscle fiber function in mechanically ventilated newborn lambs. **a** Representative examples of plots of absolute isometric force generation after exposure to calcium by diaphragm myofibrils isolated from CTL, MV, and MV+SD lambs. **b** Group mean maximal isometric specific force (normalized to cross-sectional area). **c** Group mean rate of force development (Kact). **d** Group mean rate of force redevelopment after acute shortening (Ktr). **e** Group mean rate of relaxation (Krel). *p < 0.05 for CTL (*n* = 5) versus MV (*n* = 4) or MV+SD (*n* = 6)

Figure 5a shows typical contractions produced by diaphragm myofibrils exposed to calcium in a representative example from each of the three experimental groups. The active myofibrillar force value generated by the MV and MV+SD group myofibrils was substantially lower than that by the CTL group, both in terms of its absolute value and in terms of specific force (i.e., force normalized to cross-sectional area). In this regard, the maximal specific force production by diaphragm myofibrils for all animals (Fig. 5b) was significantly reduced in the MV and MV+SD groups (by 26% and 27%, respectively) compared to the CTL group.

To determine myosin cross-bridge kinetics, the rates of force development (Kact) as well as force redevelopment following rapid shortening of fully activated myofibrils (Ktr) were measured. Both Kact (Fig. 5c) and Ktr (Fig. 5d) were significantly reduced in the MV and MV+SD groups compared to CTLs. The rate of relaxation after contraction (Krel) did not differ among the three experimental groups (Fig. 5e). Overall, there were no significant differences in any of the diaphragm contractility parameters between the MV and MV+SD animals.

## Discussion

To our knowledge, the question of whether the use of controlled MV triggers the biochemical and physiological alterations of VIDD in the neonatal diaphragm has not been addressed. Our investigation is also the first to examine whether surfactant deficiency, which is a frequent indication for the use of MV in premature infants [1, 2], further promotes the development of VIDD in neonates. The main finding of our study is that controlled MV in newborn lambs rapidly led to significant diaphragmatic dysfunction, irrespective of whether lung injury induced by surfactant deficiency was also present. Force generation normalized to fiber cross-sectional area (termed specific force), as well as the rate of force production, were significantly reduced to the same degree in MV and MV+SD diaphragms. We also evaluated several molecular pathways previously implicated as being causative of VIDD in adult animals and/or humans, including STAT3 [23, 24], the ubiquitin-proteasome and autophagy systems of proteolysis [5, 6], and oxidative stress [5, 8, 25]. Among the above mechanisms, only STAT3 was consistently found to be activated in mechanically ventilated newborn lamb diaphragms, and once again to an equal extent in the MV and MV+SD groups, although certain autophagy indices were greater in MV+SD relative to MV alone. There was no significant atrophy of either type 1 (slow-twitch) or type 2 (fast-twitch) MyHC-expressing fibers in either group.

A decrease in myofibrillar specific force suggests a reduction in the proportion of strongly bound myosin-actin cross-bridges and/or a decrease in the unitary force generated by individual cross-bridges. Reductions in force development rates (Kact and Ktr) imply that the kinetics of myosin-actin cross-bridge formation were also impaired, whereas the unchanged relaxation rate (Krel) indicates that myosin-actin dissociation was unaffected. The fact that superimposed surfactant deficiency did not alter the nature or magnitude of the contractility changes compared to MV alone supports the idea that MV rather than lung injury was likely the predominant factor driving this early diaphragmatic dysfunction. In addition, there was no apparent relationship between UPS gene expression or autophagy activation indices and the levels of diaphragm force production. Although our study did not identify the precise molecular basis of diaphragmatic contractile impairment, it may be explained by adverse post-translational modifications of myosin or other contractile proteins which have been reported in adult VIDD [26].

In previous studies of mature animals, many of the adverse consequences of MV on the diaphragm were linked to oxidative stress believed to originate primarily from mitochondria [8, 25, 27]. However, in the current study, neither of the two mechanically ventilated groups showed evidence of increased oxidative stress in the diaphragm. A rise in both reactive oxygen species and anti-oxidant defenses has been shown in lamb diaphragms within the first 24 h after birth [28], which could have obscured changes related to MV. We also cannot exclude the possibility of localized oxidative stress within subcellular compartments that were not detected in our analyses of whole muscle homogenates. For example, in adult VIDD models, oxidative modifications of specific contractile proteins, such as myosin or the sarcoplasmic reticulum calcium release channel (ryanodine receptor), have been demonstrated [26, 29]. In addition, JAK/STAT3 activation can lead to diaphragm weakness without evidence of oxidative stress, as reported in mice with cancer cachexia or myocardial infarction [13]. Although changes in IL-6 mRNA expression with mechanical ventilation did not attain statistical significance in our study, IL-6 transcript levels in the diaphragm were also found to be increased in adult VIDD and may have contributed to STAT3 activation [23, 24].

An unexpected finding was that MV alone (without surfactant deficiency) was associated with a downregulation of Atrogin1 and MuRF1, the two E3 ubiquitin ligases implicated in most forms of skeletal muscle atrophy [30]. This finding differs from studies of VIDD in adults, where Atrogin1 and/or MuRF1 were generally found to be upregulated [5, 14]. It is interesting to note that the MV group diaphragms in our study also demonstrated a major increase in the expression of SIRT1, a metabolic sensor which acts as a NAD(+)-dependent deacetylase for histone and non-histone proteins. In contrast, the MV+SD group lacked this SIRT1 upregulation and also exhibited higher transcript levels of muscle proteolysis pathway genes (E3 ubiquitin ligases and autophagy). SIRT1 overexpression is capable of reducing Atrogin1 and MuRF1 induction via FoxO transcription factors in other muscle atrophy models [31]. Therefore, we speculate that SIRT1 upregulation may have acted in a compensatory fashion to inhibit the expression of these atrophy-inducing genes in the MV group, whereas this potentially protective response was not observed in the MV+SD lambs. Further studies will be required to test this hypothesis.

There has been very little study of MV effects on the diaphragm in the pediatric context. An early study found selective diaphragm atrophy (i.e., not present in extradiaphragmatic muscles) in infants ventilated for > 12 days compared to those ventilated for < 7 days [32]. In young pigs (15–20 kg thus approximately 8–10 weeks of age), it was reported that signs of oxidative stress, atrophy, and decreased force production occurred in the diaphragm after 3 days of controlled MV [33, 34]. More recently, serial ultrasound-based measurements of diaphragm thickness also suggested progressive diaphragm atrophy in children (median age 16.5 months, range 5.5–52 months) undergoing MV for acute respiratory failure [35]. Adult animals develop VIDD with a rapidity that is inversely proportional to their body size, and we have previously hypothesized that this may be due to the higher basal metabolic rate of smaller species [36]. Since the basal metabolic rate of infants is significantly higher than in adults [37], neonates could also be subject to a more accelerated onset of VIDD. Our diaphragm myofibrillar contractility findings in newborn lambs are very similar to recently reported data from adult humans subjected to MV for a mean period of approximately 2 days [21].

There are several limitations to our study. First, the duration of the protocol was relatively short and might not accurately reflect the influence of more prolonged periods of MV and lung injury, which would usually be present in neonatal ICU patients. It is also conceivable that the impact of surfactant depletion on VIDD might differ from other forms of lung injury. Second, while muscle contractility and most biochemical outcomes did not differ between the MV and MV+SD diaphragms, there were differences in tidal volume, FiO2, PaO2, and the total duration of mechanical ventilation between these groups. These differences would have been expected to favor more severe diaphragm dysfunction in the MV+SD group which did not occur, but one cannot absolutely rule out an effect. Third, the drugs employed for sedation and analgesia, as well as the use of neuromuscular blockade to prevent spontaneous breathing efforts, could also exacerbate VIDD [38]. Although the diaphragmatic force loss in our ex vivo contractility assay cannot be attributed to persistent neuromuscular blockade or reduced central drive since it is independent of these factors, other effects of these drugs on our findings cannot be excluded.

## Conclusions

Controlled MV in neonatal full-term lambs led to the rapid onset of major diaphragm weakness that was not significantly influenced by the co-existence of lung injury caused by surfactant deficiency. Future studies are needed to determine whether different interventions which prevent VIDD in mature animals [3, 4] are able to mitigate the adverse effects of MV on the diaphragm in newborns.

## Additional file

Additional file 1: **Table S1.** Primer sequences for qPCR (DOCX 14 kb)

## Abbreviations

GAPDH: Glyceraldehyde-3-phosphate dehydrogenase
MV: Mechanical ventilation
MyHC: Myosin heavy chain
POLR2A: RNA polymerase polypeptide A
RPL19: Ribosomal protein L19
SIRT: Sirtuin
UPS: Ubiquitin-proteasome system
VIDD: Ventilator-induced diaphragmatic dysfunction
