# Supplementary material for: Mechanical ventilation causes diaphragm dysfunction in newborn lambs
Source: Crit Care. 2019 Apr 16;23:123. doi: 10.1186/s13054-019-2409-6 (PMC6469194; doi:10.1186/s13054-019-2409-6)
Supplement: Supplementary file 1 — Table S1. Primer sequences for qPCR (DOCX 14 kb) [file 13054_2019_2409_MOESM1_ESM.docx]

**Table S1. Primer sequences for qPCR**

| Gene name | Forward (5′-3′) | Reverse (5′-3′) |
| --- | --- | --- |
| Atrogin1 | AAA GTC CTT GAA GAC CAG CAA | AAG CAC AAA GGC AGG TCT GT |
| Gabarapl1 | GGGTCCCTGTGATTGTGGAG | TAAGGCGTCCTCAGGTCTCA |
| IL6 | CTGCAGTTCAGCCTGAGAG | CCCAGTGGACAGGTTTCTGA |
| LC3B | CTTCAAACAGCGCCGAACC | CCAGGACAGGAAGCTGTTTCT |
| MuRF1 | TGT GCC AAC GAC ATC TTC CA | GAT GAT GTT CTC CAC CAG CA |
| MyHC-1 | TCGTCAAGGCCACAATTTG | CTGCTGCAACACCTGGTCCT |
| MyHC-2a | AAGCCTTTTGATGCCAAGACT | TTCACCGTCACTTTCCCACC |
| MyHC-2x | CTTCGTGGCGGACCCTAAG | CAGTTACTGTCGCCCCAGCT |
| MyHC-emb | ATTAAGAGCACCCAGGACGG | TCCTCGATCCGGTCGAACTT |
| MyHC-neo | AGGCCGAGGTGGAGGAG | TCGTGTTGATCAGGCTGGTG |
| SIRT1 | CTTCTACGACGACGCGAGAG | TTTCGCACGAATGGAAACCG |
| GAPDH | CCGCATCCCTGAGACAAGAT | ACGATGTCCACTTTGCCAGT |
| POLR2A | GGCTGCTTGGAGAGATCGAG | TTCAAGGTCATCTGGGTGGC |
| RPL19 | TCGCCGGAAGGGCAGGCATA | GGCTGTGATACATGTGGGGGTC |
